# Artificial intelligence in oncology: Path to implementation

**DOI:** 10.1002/cam4.3935

**Published:** 2021-05-07

**Authors:** Isaac S. Chua, Michal Gaziel‐Yablowitz, Zfania T. Korach, Kenneth L. Kehl, Nathan A. Levitan, Yull E. Arriaga, Gretchen P. Jackson, David W. Bates, Michael Hassett

**Affiliations:** ^1^ Division of General Internal Medicine and Primary Care Department of Medicine Brigham and Women's Hospital Boston MA USA; ^2^ Department of Psychosocial Oncology and Palliative Care Dana‐Farber Cancer Institute Boston MA USA; ^3^ Harvard Medical School Boston MA USA; ^4^ Division of Population Sciences Dana‐Farber Cancer Institute Boston MA USA; ^5^ Department of Medical Oncology Dana‐Farber Cancer Institute Boston MA USA; ^6^ IBM Watson Health Cambridge MA USA; ^7^ Department of Pediatric Surgery Vanderbilt University Medical Center Nashville TN USA

**Keywords:** artificial intelligence, deep learning, machine learning, oncology

## Abstract

In recent years, the field of artificial intelligence (AI) in oncology has grown exponentially. AI solutions have been developed to tackle a variety of cancer‐related challenges. Medical institutions, hospital systems, and technology companies are developing AI tools aimed at supporting clinical decision making, increasing access to cancer care, and improving clinical efficiency while delivering safe, high‐value oncology care. AI in oncology has demonstrated accurate technical performance in image analysis, predictive analytics, and precision oncology delivery. Yet, adoption of AI tools is not widespread, and the impact of AI on patient outcomes remains uncertain. Major barriers for AI implementation in oncology include biased and heterogeneous data, data management and collection burdens, a lack of standardized research reporting, insufficient clinical validation, workflow and user‐design challenges, outdated regulatory and legal frameworks, and dynamic knowledge and data. Concrete actions that major stakeholders can take to overcome barriers to AI implementation in oncology include training and educating the oncology workforce in AI; standardizing data, model validation methods, and legal and safety regulations; funding and conducting future research; and developing, studying, and deploying AI tools through multidisciplinary collaboration.

## Graphical abstract

Artificial intelligence (AI) in oncology has demonstrated accurate technical performance in image analysis, predictive analytics, and precision oncology delivery. Yet, adoption of AI tools is not widespread and the impact of AI on patient outcomes remains uncertain. Overcoming implementation barriers for AI in oncology will require training and educating the oncology workforce in AI; standardizing datasets, research reporting, validation methods, and regulatory standards; and funding and conducting prospective clinical trials that demonstrate improvement in patient outcomes.

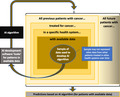

## 1 INTRODUCTION

**FIGURE 1:**
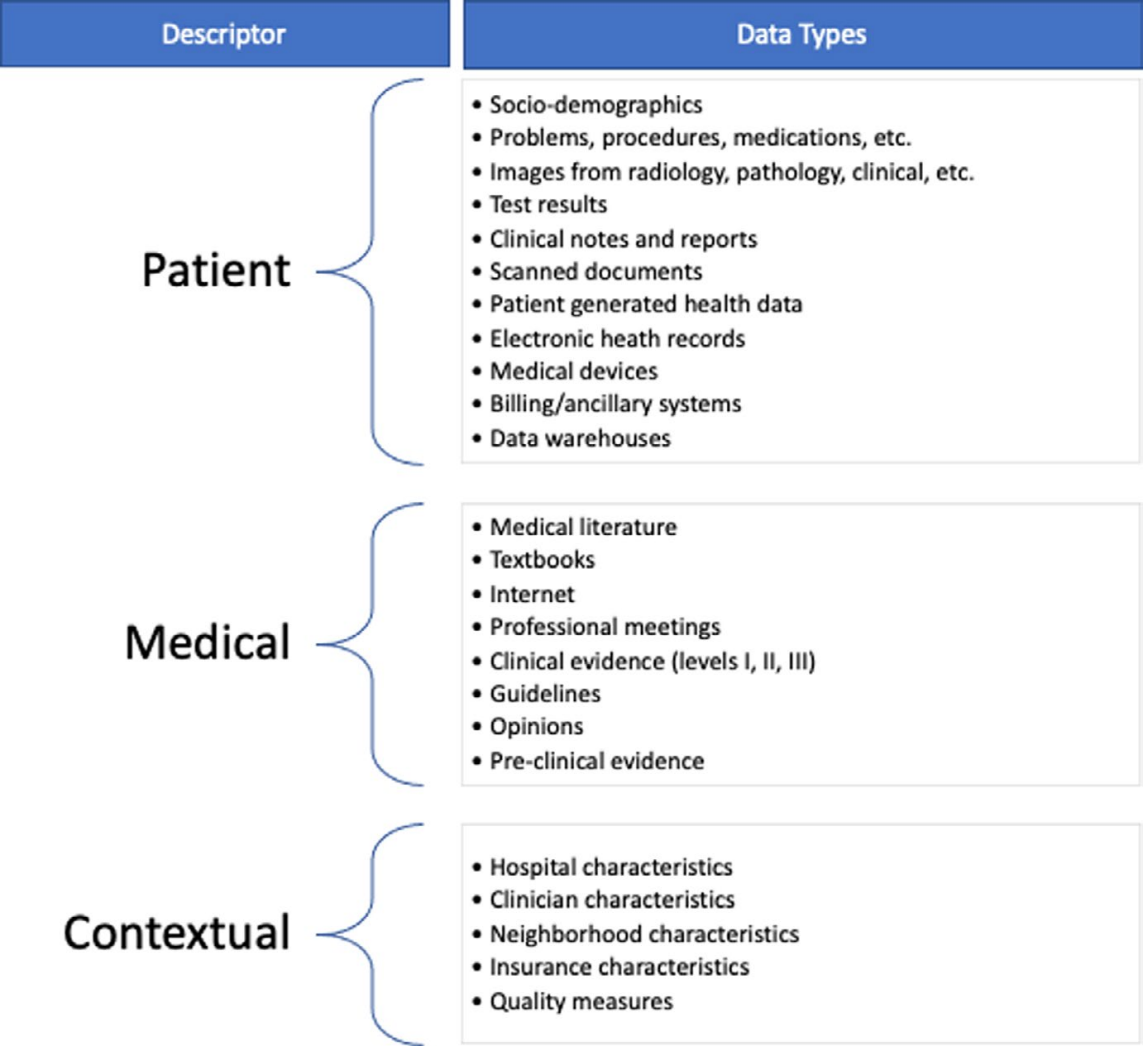
Data types and sources processed by artificial intelligence. The right column exemplifies commonly used data types that can be processed by artificial intelligence. The left column categorizes the data types into three main areas: patient, medical, and contextual

Artificial intelligence (AI) is a subfield of computer science that studies algorithms to perform tasks typically associated with human cognition.^1^ AI has been applied to problems in medicine for many years but is now experiencing accelerated adoption due to dramatic growth in large, machine‐accessible healthcare data, powerful computing systems, and innovative software technologies. AI encompasses a variety of methods (Table 1) and processes many different forms of data (Figure 1) to gain insights that inform health care and its delivery. Similar to humans, AI can use existing knowledge and experience to recognize patterns in data; but unlike humans, AI can synthesize large amounts of complicated and disparate data quickly without being prone to fatigue.

**TABLE 1:** Artificial intelligence and data science terminologies

Terms	Definition
Machine learning	Algorithms and models which machines can use to learn without explicit instructions.^1^
Supervised learning	Machine learning that is based on input–output pairs.^1^
Unsupervised learning	Machine learning that proceeds without direction from a human, targeted at predicting outputs.^1^
Deep learning	A subset of machine learning that generally uses neural networks.^1^
Natural language processing	Machine learning specifically to understand, interpret, or manipulate human language.
Computer vision	Machine learning that trains computers to interpret and understand the visual world.
Knowledge representation	A surrogate that is used to enable an entity to determine consequences by thinking rather than acting and is a set of ontological commitments, a fragmentary theory of intelligent reasoning, and a medium for pragmatically efficient computation and human expression.^87^
Ontology	Controlled terminology invoking formal semantic relationships between and among concepts, manifested as a type of description logic, which is a subset of first‐order predicate logic, chosen to accommodate computational tractability.^88^
Fast Healthcare Interoperability Resources (FHIR)	Standard for exchanging healthcare information electronically created by Health Level Seven International (HL7), a not‐for‐profit, American National Standards Institute‐accredited standards developing organization.^78^
Minimal Common Oncology Data Elements (mCODE)	A collaboration between the American Society of Clinical Oncology, Inc., CancerLinQ LLC, and MITRE to identify minimal cancer data elements that are essential for analyzing treatments across patients via their electronic health records.^54^
Informatics	The science of how to use data, information, and knowledge to improve human health and the delivery of healthcare services.^89^

AI algorithms have the potential to transform health and healthcare delivery by helping solve complex problems, such as improving quality of life, prolonging survival, maximizing safety, and increasing value. An AI algorithm consists of rules or processes developed using AI methods that are applied to a specific scenario to parse, learn from, or make informed decisions about data. They can be developed using rule‐based approaches that require human input (i.e., supervised) or statistical approaches that do not require human input (i.e., unsupervised) (Table 1). Common applications in health care include identifying conditions, events, risk factors, associations, and patterns of difference or similarity, which can be used to support clinical decision making, enable population health management, reduce administrative burdens, increase efficiency, and facilitate discovery.^2^

The opportunity for AI to impact oncology‐related problems is great because oncology has become exceptionally complex. However, few AI tools have yet had a significant and widespread impact in oncology.^3^ The goals of this manuscript are to review current and future AI‐based solutions for oncology problems; to discuss barriers to implementation of impactful, cancer‐focused AI solutions; and to propose steps that will help foster the development and deployment of AI tools in routine clinical practice. Although our discussion regarding the solutions, barriers, and opportunities of AI focuses on oncology, many of the issues we discuss are relevant for other fields of medicine.

## 2 OVERVIEW

Several features of oncology enable AI to have a substantial impact. First, the societal burden of cancer is great. More than 1.8 million people are diagnosed and approximately 600,000 people die from cancer each year in the United States.^4^ Second, cancer care is extremely expensive and costs are increasing rapidly, with an estimated annual U.S. spending on cancer exceeding $140 billion and reaching $173 billion by 2020.^5^ Third, optimal treatment planning requires interpreting and synthesizing large amounts of complex data from different sources, including pathology, laboratory, radiology, and advanced molecular diagnostics. Although these characteristics make oncology ripe for the application of AI solutions, they also introduce significant obstacles to their development.

First, oncology treatment planning is challenging because cancer includes many distinct conditions, each with unique and complex patterns of care; and cancer treatments often have narrow therapeutic indices with serious adverse effects. Therefore, balancing harms and benefits is a constant struggle. Second, treatment paradigms are changing rapidly due to the pace of scientific discovery. Third, cancer care is multidisciplinary—requiring coordinated input from multiple stakeholders (e.g., medical oncologists, radiation oncologists, surgeons, primary care providers, patients, caregivers, and others). Fourth, cancer care is inherently multisite—with services being delivered at inpatient hospitals, outpatient treatment centers, ambulatory clinics, and other locations that often have different and siloed methods of recording, storing, and transmitting data. Finally, cancer care is typically administered longitudinally across disparate settings, including initial/adjuvant/curative/maintenance care, survivorship, relapse/recurrence, and end‐of‐life care. Although these complexities create substantial barriers for machines, they also create large hurdles for humans and represent opportunities where AI algorithms could have significant impact on the quality and value of cancer care, especially in communities that lack oncology subspecialists.^6^, ^7^

## 3 APPLICATIONS

To date, some of the most promising work on AI in oncology is taking place within the area of cancer imaging, specifically digital pathology, radiographic imaging, and clinical photographs.^8^ In digital pathology, AI has been applied to both low‐ and high‐level image processing and classification tasks (e.g., tumor detection and segmentation as well as predicting disease diagnosis and treatment responses based on image patterns) to automate time‐consuming tasks and to improve diagnostic accuracy.^9^, ^10^, ^11^, ^12^ In radiology, multiple evaluations have demonstrated that AI tools can differentiate between high‐ and low‐risk lesions on a wide variety of imaging modalities.^13^, ^14^, ^15^, ^16^, ^17^, ^18^, ^19^, ^20^, ^21^ Integration of radiographic imaging with other sources of data (e.g., clinical features and genetic/biochemical markers) to risk stratify image‐detected lesions already exists and will likely be more commonplace in the future.^22^, ^23^ AI is also being used to help improve diagnostic accuracy and reduce diagnostic uncertainty among dermatologic and gastrointestinal malignancies on clinical imaging.^24^, ^25^, ^26^, ^27^, ^28^

AI can provide accurate estimates of a patient's risk for experiencing a host of outcomes, including rehospitalization, cancer recurrence, treatment response, treatment toxicity, and mortality.^29^, ^30^, ^31^, ^32^, ^33^, ^34^ Enhanced predictions may offer potential advantages, including facilitating treatment planning, guiding population management efforts, and fostering discussions about goals of care.^35^, ^36^ Predictive analytics may aid the delivery of oncology treatments to populations that are disadvantaged or underrepresented in clinical trials for whom it is challenging to apply evidence‐based medicine.^37^ For example, among older adults, AI predictive analytics may help oncologists anticipate problems that are not detected during a comprehensive geriatric assessment or identify risk factors for chemotherapy toxicity that are overlooked in daily practice.^38^, ^39^, ^40^

Another application of AI involves precision oncology. The rapid growth of genomic tumor characterization has led to the development of computational methods to aid interpretation of these data and to foster the delivery of precision oncology.^41^ For example, AI can facilitate tumor genomic data analysis by reporting potentially actionable variants on tumor next‐generation sequencing assays. One AI‐based system produced results more rapidly and accurately than humans and facilitated identification of eligibility for participation in biomarker‐selected clinical trials.^42^, ^43^ Machine learning (ML) (Table 1) can also predict tumor type from a targeted panel of DNA sequence data and thereby support the selection of more appropriate therapy.^44^ Additionally, ML and deep learning (Table 1) have been shown to augment the detection ability and accuracy of liquid biopsies.^45^, ^46^, ^47^ As utilization of liquid biopsies become more widespread,^48^, ^49^ AI‐based tools may become invaluable to clinicians who must appropriately order and interpret these complex tests.

**FIGURE 2:**
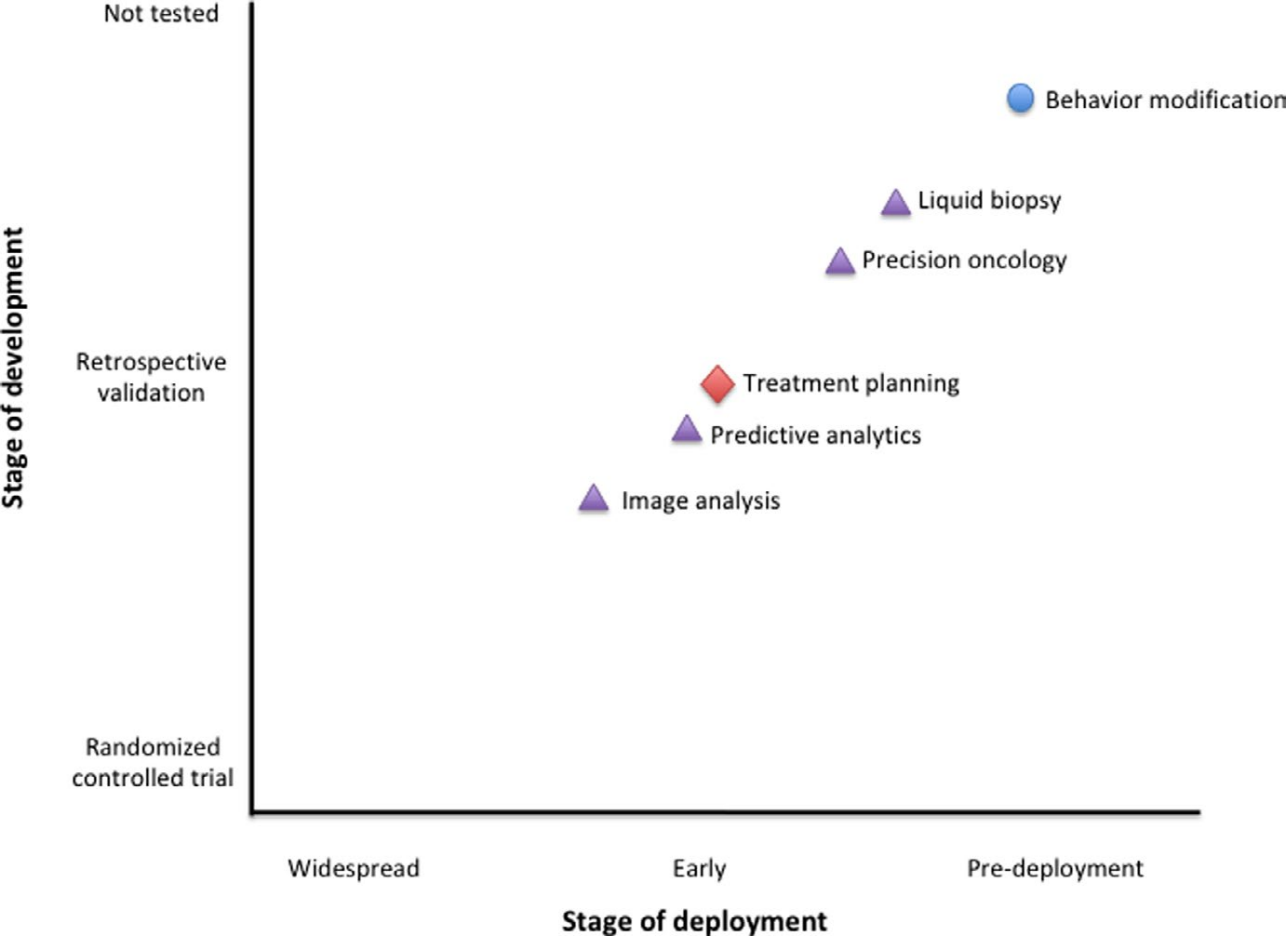
Stage of development and deployment among applications of artificial intelligence in oncology. The location of a topic represents the farthest that topic has come in its development, not necessarily the point in development where all solutions in that topic area have reached. Each topic's shape represents its application within the levels of cancer prevention (circle = primary, triangle = secondary [or tertiary], diamond = tertiary)

There are substantial opportunities for AI to impact primary cancer prevention as well (Figure 2). Approximately half of cancers could be prevented by applying knowledge we currently possess about cancer risk mitigation.^50^ Behavioral modification is a key to cancer prevention, but behavioral change interventions remain underutilized.^51^ AI can bridge this gap by helping policymakers and clinicians efficiently synthesize, interpret, and disseminate evidence for cancer prevention. One research group is creating an ontology (Table 1) of behavioral change evaluations; training an automated feature extraction system to annotate evaluation reports using this ontology; developing ML models to predict effect sizes for combinations of behaviors, interventions, populations, and settings; and building interfaces for interrogating and updating the knowledge base.^52^ In the future, this technology could help clinicians deliver precision prevention by recommending interventions that incorporate a patient's unique biologic, behavioral, and socioeconomic characteristics.^53^

## 4 SPECIFIC BARRIERS

Although the promise for AI applications in oncology remains great, the benefits for oncology still seem far off. Important challenges and questions remain, including the burden of data standardization, collection, and management; the bias inherent to training data sets; the lack of robust reporting standards; the relative scarcity of prospective clinical validation studies; user‐design and workflow implementation challenges; outdated regulatory and legal frameworks that surround AI; and the exponential growth of knowledge and dynamic data.

### 4.1 Burdens of data standardization collection and management

Healthcare data are often recorded and stored in highly heterogeneous, idiosyncratic, and unstructured ways. Therefore, AI algorithms developed using one system's data may perform less well if applied to a different system's data. Standardization of terminology and data collection will increase the proportion of electronic health record (EHR) data that are ontology‐integrated; this is essential for AI to have a meaningful impact in oncology. Efforts, such as the minimum Common Oncology Data Elements (mCODE) initiative (Table 1), are addressing these challenges,^54^ but deploying solutions widely and consistently will take substantial effort. Ideally, data standardization should occur before algorithm development, when information is first collected. Patient‐reported outcome measures (PROMs) represent an ideal mechanism to collect standardized data early in the process directly from the patient. In oncology, PROMs are already being used to identify early signs of patient distress and to evaluate quality of care; however, they face some of the same implementation challenges discussed above.^55^ In particular, the demand to collect more data could exacerbate clinician burnout. Some AI solutions rely on data from multiple sources (e.g., patient‐level EHR data and medical knowledge databases), which further exacerbates the data collection and management burdens. The administrative and financial costs of managing and maintaining disparate data types are substantial and may be prohibitive for smaller practices.

### 4.2 Biased training data

**FIGURE 3:**
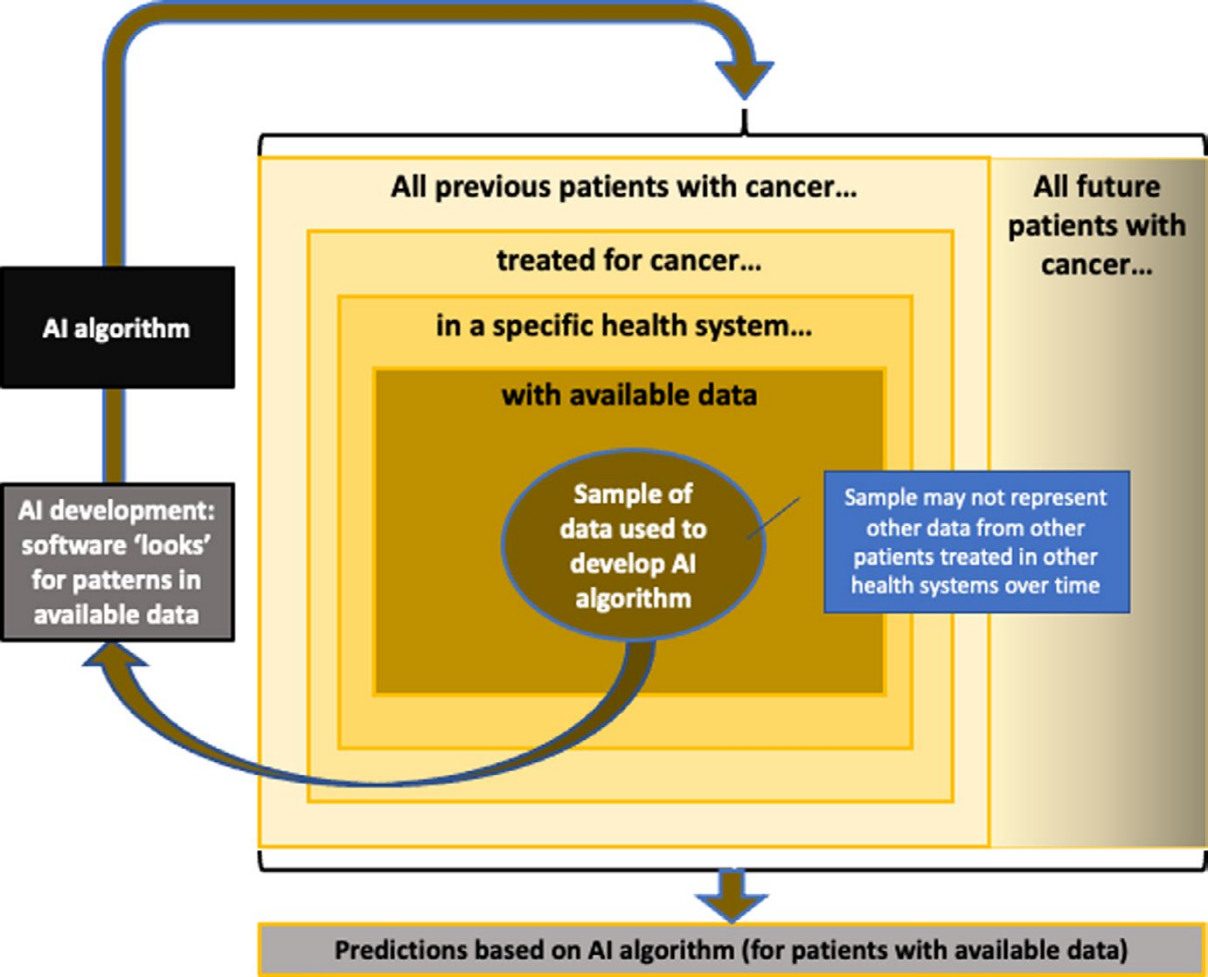
Statistical biases associated with artificial intelligence (AI) algorithm predictions. AI‐based tools look for patterns of association in the data made available to them; they do not establish causation. The sample of data used to develop an AI algorithm may not represent data from other patients treated in other health systems over time. For example, if most of the data used to develop an AI algorithm came from patients <65 years old treated before 2018, then an AI algorithm may not provide reliable estimates for patients >65 years old treated after 2020

AI, in its current form, is essentially focused on pattern recognition. Therefore, any pattern embedded within the data used to develop a model will be propagated to the predictions generated by that model. This could be problematic if the data used to develop the model differ systematically from the data to which the model is applied. Figure 3 illustrates how biased sampling, a form of statistical bias,^56^ may lead to inaccurate predictions. For example, when clinical trial data serve as the foundation for an AI algorithm, traditionally underrepresented populations (e.g., adolescents and young adults, women, ethnic minorities, the elderly)^37^, ^57^ within the data set may affect the ability for AI to generate an accurate recommendation for these particular subgroups. Ensuring representative sampling across time (i.e., recently vs. historically treated patients) and data sources (e.g., medical record data from different health systems) is also important to prevent this type of bias.

**FIGURE 4:**
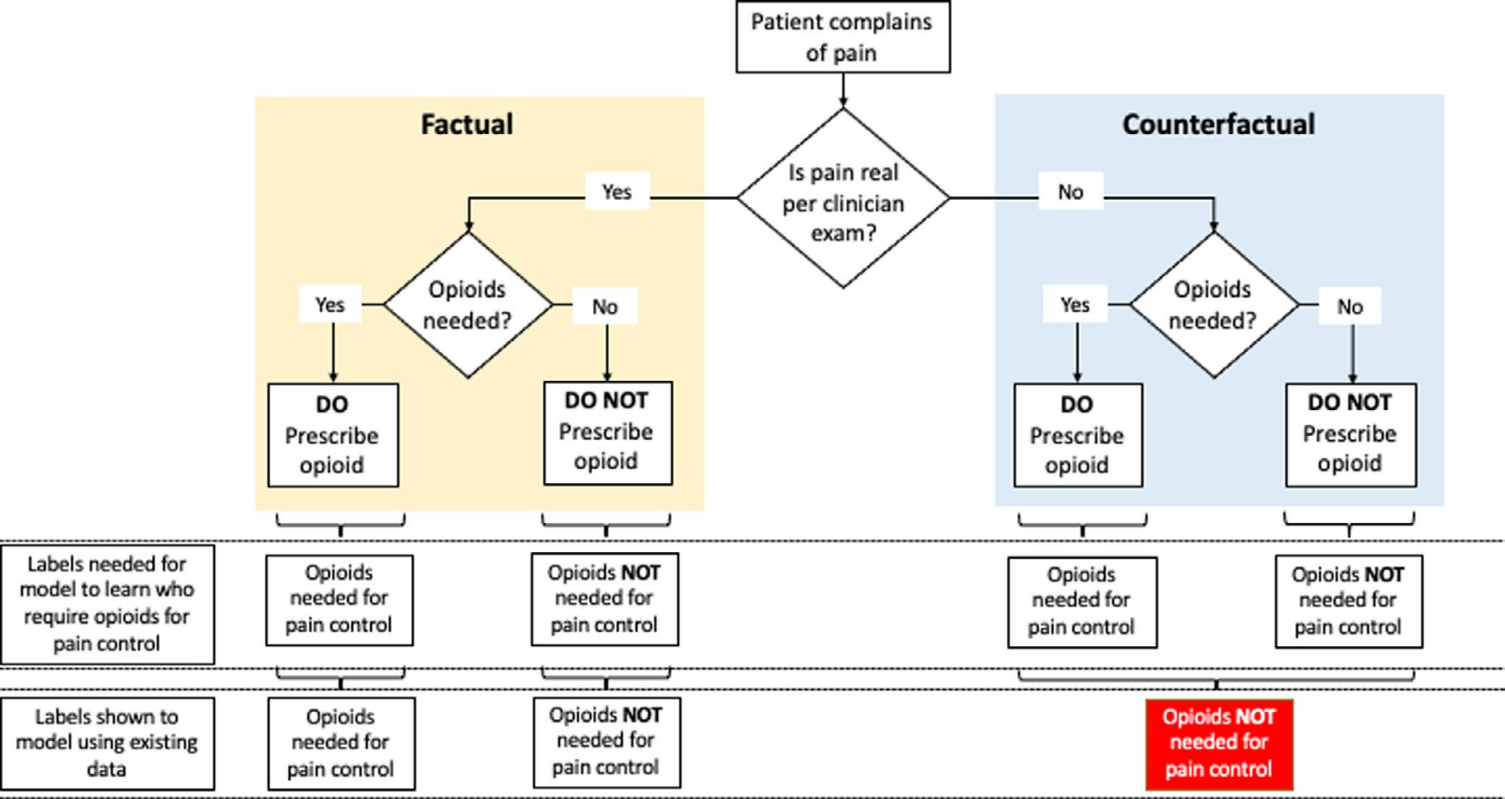
Social biases associated with artificial intelligence algorithm predictions. This figure depicts the gap between what we need to show the model (i.e., both factual and counterfactual scenarios) versus what happens when machine learning (ML) is trained on existing data. In this example, an ML model is used to identify oncology patients who require opioids for pain management. When using existing data (i.e., secondary use of data collected as part of routine work), the data reflect not only the association between the patient's condition and opioid prescribing, but rather it reflects this association conditioned on the staff's determination if the patient's complaint of pain is legitimate or not. If the staff's decisions are not uniform (e.g., biased by demographics), then some of the patients who were not prescribed opioids will have the wrong label (“opioids not needed for pain control”), whereas they should have had the label (“opioids needed for pain control”). Therefore, the model will be shown the wrong labels and will learn an erroneous pattern

AI is also vulnerable to social bias, which is when inequities in healthcare delivery systematically lead to suboptimal outcomes for certain groups.^56^ For example, if an AI model were developed to assist with pain control, the resulting algorithm could potentially provide suboptimal predictions for black patients (Figure 4). Here, the issue is not that black patients were excluded from the training data set, but rather clinicians have historically undertreated pain among black patients due to unconscious biases.^58^, ^59^ Therefore, a pattern of behavior that is intrinsic to a training data set can be propagated into the future when the model is applied in a clinical setting. Because AI uses latent or obscure representations as independent variables, it can be hard to explain why predictions are made and difficult to gauge when predictions do not make sense. Consequently, using AI for predictive tasks risks propagating erroneously learned patterns into recommendations and clinical practice.

Increased inclusion of underrepresented groups in the training data will be necessary to ensure prediction accuracy across all populations. However, this will take time and will require multilevel interventions that influence attitudes, communication, and actions at the patient, oncologist, and interpersonal levels.^60^ Meanwhile, AI‐based solutions will need to address the inextricable biases that are often present in training data sets. Computational methods that detect, understand, and mitigate preexisting bias in a training data set are being developed.^61^, ^62^ Potential solutions could involve developing methods to estimate the bias of a specific data set and establishing standards that determine when the bias is sufficiently concerning to question the use of that particular data set for algorithm training or as a target for deployment.

### 4.3 Lack of research reporting standards and prospective clinical validation

The absence of AI reporting standards has contributed to a reproducibility crisis, which could limit the widespread adoption of AI.^63^ Because AI algorithms, especially deep learning methods, are sensitive to subtle nuances in the data that cannot be identified, lack of reproducibility is a real possibility that could be hard to overcome. Increasing reporting requirements regarding an algorithm's source code and training conditions could help address this problem, but transparency could raise concerns regarding intellectual property and competitive advantage for companies that invest in AI.

Additionally, few published studies in oncology have compared the effects of AI or AI‐assisted interventions with the standard of care on patient outcomes.^18^, ^64^ Consequently, the extent to which AI will impact patient outcomes and cost remains uncertain. Establishing clinical validity and cost‐effectiveness would require conducting randomized controlled trials (RCTs) with patient‐relevant endpoints. However, RCTs are expensive and time‐consuming, and the design will need to consider the multiple dimensions of uncertainty when evaluating AI interventions, especially for those that augment rather than replace human activity.^65^

### 4.4 Workflow and user‐design challenges

Successful implementation of AI must address the sociotechnical challenges encountered within complex adaptive healthcare systems.^66^ To encourage widespread adoption, AI‐based solutions must be integrated seamlessly into the clinician's workflow, be intuitive to use, and provide value to the user. This is a greater challenge for some applications of AI than others. Although not all AI applications that analyze data need to be delivered through interactive environments for use by clinicians, key factors for adoption include having explainable and actionable output and being well integrated into clinical workflows. Clinical decision support systems (CDSS) for oncologists, however, often require interfaces that are more interactive and explanatory. To the extent that AI‐based solutions can be adaptive or multifaceted, the dynamic aspects of these solutions must be apparent to the end‐user. For example, if AI‐based CDSS adapt longitudinally to changes that occur during therapy (e.g., anatomic and physiologic changes to tumor and surrounding normal tissue during radiotherapy), these facets must be transparent to the clinician.^67^

Clinicians must understand the basis of a recommendation, find it relevant, and trust the evidence on which the algorithm is based.^68^ Although algorithm output should always strive to be comprehensible, the threshold for understanding AI output will vary depending on the use case and AI method utilized. For example, for direct patient care (e.g., an oncologist using AI to predict a patient's treatment mortality risk), the threshold for output comprehensibility is high as the results will greatly influence clinical decision making, especially because clinician experience with and trust in AI‐based solutions are currently low. In this circumstance, supervised ML (Table 1), which predicts a specific output (e.g., mortality risk) using inputs (e.g., patient data), would be advisable because a gold standard output is required. However, in circumstances, where some uncertainty is expected or considered reasonable, unsupervised ML (Table 1), where there is no target output to predict, may be appropriate.

Even if AI predictions are accurate and comprehensible, the desired improvement will not result if users do not take appropriate action. Designing AI tools that incorporate behavioral economics principles and support positive behavior change (e.g., setting default options or behavioral nudges) for clinicians and patients may help.^69^, ^70^ However, designing AI tools that optimize user adoption must be counterbalanced by both ethical and safety considerations, including minimizing automation complacency, which is when a user accepts the system's recommendations as infallible or using the recommendations to confirm initial assumptions without critically considering alternatives.^71^

### 4.5 Regulatory and legal frameworks

How legal and regulatory frameworks should guide the development and deployment of AI in health care is a topic of great debate.^72^ The legal and regulatory challenges facing AI‐based decision support tools, which have bedeviled decision support systems for many years and are not unique to oncology, will have to be addressed for these tools to have any chance to become part of routine clinical practice. Legally, the dearth of case law involving medical AI makes navigation of medical liability complex. Current tort law may incentivize physicians to minimize the potential value of AI by using it as a confirmatory tool rather than as a way to improve care.^73^ Moreover, many physicians are concerned about the level of patient safety and their legal responsibility for diagnostic errors made by AI.^74^ Consequently, ambiguous malpractice liability policies may remain a significant barrier to clinicians proactively adopting AI into routine practice.

### 4.6 Dynamic knowledge and data

Regardless of the regulatory framework in place, algorithms implemented in a real‐world oncology setting will need to keep pace with the exponential growth in cancer research. They will also have to account for dynamic changes in source data, which may be precipitated by evolving data standards and ontologies; modernizing electronic health record systems; changing documentation and reimbursement policies; or novel diagnostic technologies. Methods for repeatedly evaluating algorithm accuracy or updating algorithms when their performance begins to drift secondary to shifts in underlying data distributions must be developed. Certain algorithms may also need to have an automatic expiration, which would prompt reevaluation after a defined period of time.^75^

## 5 NEXT STEPS

The challenges facing AI in oncology are formidable and span the entire ecosystem of oncology care. Yet, these challenges are surmountable and can be addressed methodically and systematically (Table 2). We recommend the following actions that major oncology stakeholders can take to foster the development and deployment of AI tools in routine clinical care.

**TABLE 2:** Next steps toward artificial intelligence (AI) implementation in oncology

Training and educating the oncology workforce
Develop educational modules for practicing oncologists who address interpretation and application of AI‐based tools
Incorporate formal training on basics of medical informatics and implementation science in fellowship curricula
Expand and stimulate career tracks focused on informatics applied to oncology
Train health system administrators and information system leaders regarding the demands and impacts of AI‐based solutions.
Standardizing data, research and validation methods, and regulatory standards
Develop and expand use of standard oncology terminologies and ontologies
Develop standards that foster systematic evaluation of performance of AI‐based tools
Establish consensus regarding regulatory and legal frameworks for AI‐based tools
Funding and conducting future research
Conduct research that fosters development of optimal methods for balancing competing aspects of fairness
Conduct randomized controlled trials that test the impact of AI‐based tools on patient survival, quality of life, and cost‐effectiveness.
Conduct implementation science research that study optimal methods for deploying AI‐based tools in routine care settings.
Conduct behavioral research on how data visualizations of AI‐based recommendations affect clinical decision‐making in oncology
Developing, studying, and deploying AI tools through multidisciplinary collaboration
Increase engagement with clinical information system vendors and EHR companies
Support partnerships between informatics companies, academia, professional societies, health systems, and community‐based practices to enable widespread deployment

First, we recommend training and educating current and future oncology workforce and leadership to become proficient adopters of AI‐based CDSS and to stimulate and expand oncology career tracks in informatics (Table 1).^76^ Oncology professional societies should develop AI education modules for oncologists and formal training should be included in fellowship. If possible, institutional leaders and academic deans should include engineers and informaticians in their faculty to assist learners in their understanding and use of AI. Fostering close collaboration between oncologists‐in‐training and nonclinical experts in the development of AI applications will also push research regarding assessment, understanding, and application of data in the care of patients.^77^ However, most clinicians do not need to become informaticians or computer scientists to use AI tools in practice. Instead, they should understand AI at a high level; specifically, how AI applications operate, what the pitfalls are, and what science is needed to show that they work. Additionally, health system administrators and leaders should receive training because their understanding will support prioritization of AI‐based tools relative to other business needs, facilitating identification and selection of AI‐based solutions most likely to impact business processes and clinical outcomes.

Second, efforts to standardize oncology terminology should continue, which would allow for reports to consist largely of structured data elements. Fast Healthcare Interoperability Resources (FHIR) standards (Table 1) and the mCODE initiative are significant advances to facilitate interoperability of oncology patient data.^54^, ^78^ These efforts should be further expanded toward other areas of oncology, including genomics‐ and patient‐reported outcomes, whose integration and analysis by AI have been hindered by lack of data standardization and limited EHR integration. AI developers should also actively engage with EHR vendors to facilitate access to data for AI initiatives within and across institutions and to enable incorporation of AI‐based strategies into clinical workflow while minimizing data collection and management burdens.

Third, formalizing standards for external and continuous validation of AI models and increasing research on algorithm fairness are needed to minimize AI bias in oncology. As noted above, using nonrandomized, real‐world, historical data could introduce bias into the algorithm, which could affect the validity of predictive modeling.^79^ At their core, data‐driven AI methods (e.g., ML and deep learning) recognize the patterns in the training data, and when presented with real‐world data, such methods will propagate any bias already present in the data. Therefore, standards and guidelines for the validation of AI systems are needed to promote clear and uniform measures of their accuracy and correctness. However, even in the absence of biases in the data, fairness remains a challenge. Differences in disease patterns may preclude equal accuracy of models across different groups, and algorithm developers will have to trade off competing aspects of fairness (e.g., types of inequality).^80^ Because simply removing group membership from the data does not guarantee fairness,^81^ further research on unfairness sources, mitigation methods, and testing standards are needed to facilitate fairness in practical applications of AI in oncology.^79^

Fourth, establishing consensus around a robust yet sensible regulatory framework that fairly assigns liability due to AI‐related error while ensuring an acceptable level of quality and safety for AI tools is necessary to foster trust with both oncologists and patients. However, updating preexisting legislative frameworks to regulate AI in healthcare will likely be insufficient. Lawmakers should obtain multidisciplinary counsel from ethicists, computer scientists and informaticians, clinicians, patients, professional societies, and health technology companies to construct a new regulatory framework that takes into consideration AI's self‐learning characteristic. The US Food and Drug Administration recently published an action plan that embraces this multidisciplinary stakeholder approach to promote good ML practice, incorporate transparency to users, develop regulatory science methods related to algorithm bias and robustness, and monitor real‐world performance.^82^

Fifth, standardizing research reporting and conducting prospective RCTs that demonstrate improvement in traditional patient outcomes are essential for AI adoption. Because AI systems are costly, healthcare leadership will be more willing to adopt AI systems when there is evidence of improved patient outcomes (e.g., survival, quality of life, and cost‐effectiveness). Researchers have recently published consensus‐based guidelines for evaluating and reporting clinical trials for AI interventions.^83^, ^84^ Others have recommended a phased approach akin to the phases of clinical trials required for the approval of drugs and devices.^85^ Careful adherence to such a robust and sequential evaluation can avoid common pitfalls in AI implementation in clinical settings. However, this approach is resource intensive and will likely require partnerships between academia, community‐based practices, public agencies, and industries. Moreover, self‐learning AI tools are dynamic and their safety and efficacy profile will likely change over time, which will make diligent and frequent postmarket safety monitoring especially important.

Finally, implementation science and behavioral research is needed to understand how to optimize workflow integration of AI in oncology and how data visualizations of AI‐based recommendations affect clinical decision‐making around cancer treatment, respectively. Current AI‐based CDSS available to oncologists are add‐on tools that interrupt clinician workflow and are time‐consuming to use. To achieve widespread adoption, AI‐based tools should integrate seamlessly into clinician workflows and be platform agnostic, including but not limited to the EHR, tumor board platforms, and payer precertification systems. Also, decision‐making about cancer treatments is a high stakes endeavor that is inherently complex and riddled with human biases and heuristics.^86^ Consequently, AI designers should understand how visualizations of AI recommendations affect clinical decision‐making and incorporate patient priorities to better ensure that its recommendations are presented in a way that is ethical, evidence‐based, and patient‐centered.

## 6 CONCLUSION

The inherent organizational complexity of cancer care delivery, the need to interpret and synthesize vast amounts of data from different sources, the narrow therapeutic window of treatment, and the heterogeneity of cancer make oncology a challenging, yet ideal area to develop and implement AI tools. To date, AI in oncology has demonstrated accurate technical performance in image analysis, predictive analytics, and precision oncology delivery and may potentially be used to facilitate primary cancer prevention in the future. However, additional research is needed to understand AI's effect on patient outcomes and cost. Additionally, barriers to AI implementation in oncology are formidable and span the entire ecosystem of oncology care. The level of effort needed to train and educate the oncology workforce; standardize data sets, research reporting, validation methods, and regulatory standards; and fund and conduct future research will require an enormous multidisciplinary effort. Therefore, establishing partnerships across healthcare systems, academia, industry, and public agencies may be essential to AI implementation in the era of big data in oncology.

## CONFLICT OF INTEREST

Except I.S.C. and M.H., all other coauthors have declared no conflict of interests.

## AUTHOR CONTRIBUTIONS

Concept and design: all authors; Drafting of the manuscript: I.S.C. and M.H.; Critical revision of the manuscript for important intellectual content: all authors; Obtained funding: G.P.J., D.W.B.; Administrative, technical, or material support: G.P.J., D.W.B.; Supervision: G.P.J., D.W.B., and M.H. We did not seek approval from Mass General Brigham institutional review board or ethics committee prior to commencing this study because this manuscript involves no original research and is a commentary/review. Data sharing not applicable to this article as no data sets were generated or analyzed in this review.
